# Identification of Farmland Bird Indicator Species for Practitioner Monitoring in the United Kingdom

**DOI:** 10.1002/ece3.72380

**Published:** 2025-11-16

**Authors:** N. M. McHugh, E. R. Ness, R. Nichols, G. Banks, D. Strong, M. W. Young, A. Zuta, G. S. Begg

**Affiliations:** ^1^ Burgate Manor Game and Wildlife Conservation Trust Fordingbridge UK; ^2^ The James Hutton Institute Dundee UK; ^3^ Independent Contractor Collieston UK

**Keywords:** agroecology, biodiversity, citizen science, farmer cluster, farmland specialist, flagship species

## Abstract

Agricultural intensification has resulted in bird population declines on farmland, with farmland bird specialists affected the most due their reliance on more traditional farming methods. Current voluntary breeding bird monitoring schemes across Europe require the observer to identify many species by sight and/or sound and often use complex survey methods and techniques, which might deter individuals from taking part. Indicator species provide a solution whereby only a few ‘flagship’ species need to be known to the observer, and these species indicate the status of the rest of the species within that taxon. Our aim was to determine if indicator species could be identified that correlated with the diversity or abundance of different bird communities present on farmland. We used breeding bird survey data collected on two Farmer Clusters, one in England and one in Scotland between 2021 and 2024. Using the 2021–2023 data, NMDS visualisation and further GLMM testing, several species were identified as potential indicator species. Corn Bunting, Linnet and Skylark were the species most strongly correlated with specialist farmland species' abundance, with Linnet showing a significant positive relationship against specialist farmland abundance and richness in both Farmer Clusters. In both Farmer Clusters, Goldfinch showed a positive significant relationship with total bird community species richness and abundance. The reliability of these indicator species was then tested against the 2024 data. Corn Bunting, Linnet and Skylark were again found to be significant when tested against specialist farmland abundance; while in England, Goldfinch and Stock Dove were significant when tested against total bird abundance. The farmland bird indicators identified here provide a much simpler list of species for a practitioner to learn to identify and survey and allow the surveyor to capture the overall diversity of farmland birds but also the status of the specialist communities.

## Graphical abstract

Current farmland bird monitoring schemes are often too complex for widespread participation. Using breeding bird data from two UK Farmer Clusters, we identified indicator species strongly correlated with specialist farmland birds and total bird communities, including Corn Bunting, Linnet, Skylark, Goldfinch and Stock Dove. Combined, these indicators offer a simplified yet effective tool for monitoring general bird biodiversity and specialist populations.

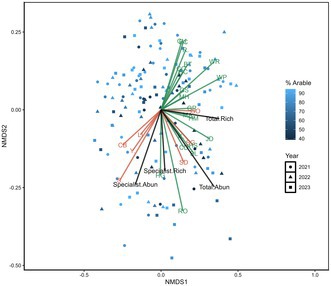

## 1 Introduction

Traditionally, farmed landscapes across Europe were characterised by complex mosaics of low input crops which maintained high biodiversity levels (Emmerson et al. 2016; Stoate et al. 2001). Due to increased demands for food and fodder, these agricultural landscapes have undergone substantial changes since the 1950s, resulting in the intensification of farming techniques and landscape homogenisation (Stoate et al. 2009). This placed increased pressure on farmland biodiversity (Reidsma et al. 2006; Tilman et al. 2001), which has seen extensive declines across a range of taxa (Abudulai et al. 2022; Abdi et al. 2021; Banerjee et al. 2024), including farmland birds (Rigal et al. 2023).

Birds are integral components of terrestrial ecosystems, contributing to pest control, seed dispersal and overall ecosystem functioning (Sekercioglu 2012; Bradbury et al. 2010). Among the bird species which have suffered severe declines as a result of agricultural intensification are specialist farmland birds (Filippi‐Codaccioni et al. 2010), a group of species which typically rely on resources provided through traditional farming practices for all or part of their life cycle, for example, Corn Bunting (*Emberiza calandra*) and Northern Lapwing *Vanellus vanellus* , hereafter Lapwing (Siriwardena, Baillie, and Wilson 1998; Sharps et al. 2023). The conservation of this suite of species has been the target of numerous government Agri‐Environment Schemes (AES), where farmers are paid in exchange for implementing specific conservation practices, for example, field margins and cover crops (Vickery et al. 2004). Therefore, monitoring these specialist farmland species is vital to track their population sizes and ranges.

Across Europe, bird monitoring initiatives dedicated to monitoring common species during the breeding season commenced in the 1960s in the United Kingdom and 1970s in Sweden (Wretenberg et al. 2006). Today around 15,000 volunteer, amateur birdwatchers, across 28 countries in Europe, use standardised protocols to contribute towards national‐level bird monitoring schemes (Brlík et al. 2021). The substantial involvement of volunteers and citizen scientists facilitates data collection on a scale otherwise unattainable. Both the standardised annual counts and observations recorded through citizen science projects generate crucial data for estimating population trends and determining the conservation status of farmland bird species (Kobori et al. 2016; Greenwood 2007; Jiguet et al. 2012; Hall et al. 2021; Kelling et al. 2019). Participating in a breeding bird survey, however, can be challenging for novice birdwatchers as the methods employed are often complicated and require a high level of skill, for example, identifying a wide range of birds to species by sight or sound. This may limit their appeal and application, excluding potentially valuable participants such as farmers.

There has been a growing appreciation of the role of indicator species (a species whose presence, absence or abundance reflects specific local environmental conditions) in monitoring and assessing farmland (Bucher et al. 2019; Oñate et al. 2000; Gregory et al. 2019; Husby et al. 2021). Indicator species are often more easily recognised by novice surveyors or practitioners (individuals working in the landscape such as farmers and land managers). Additionally, using a narrower range of species in monitoring programmes reduces expert surveyor costs and allows communication to focus on specific ‘flagship’ species whose conservation provides wider benefits to overall ecosystem health (Bucher et al. 2019; Tiede et al. 2017). Certain bird species have emerged as potential farmland bird indicators, with designation of indicator species depending on the specific goals and focus of monitoring programmes and research studies. For example, specialist species including Grey Partridge *Perdix perdix* , Yellowhammer *Emberiza citrinella* and Corn Bunting have all been cited as indicators of healthy arable farmland ecosystems in a UK context (Szymkowiak et al. 2014; Beier and Jánoska 2023). However, there is also a need to identify bird species that act as indicators of diverse bird populations, both wider bird diversity, or of specific suites of species.

Drawing on extensive field data collected through the FRAMEwork project (Taskscape 2025), a multi‐institute, pan‐European project assessing the impacts of Farmer Clusters on farmland biodiversity, possible bird indicator species for lowland mixed farmland are identified. Their suitability as indicator species is assessed by evaluating relationships between these species and measures of specialist farmland bird abundance and richness, as well as their relationships with the wider bird population (i.e., the entire community of birds present on farmland) in terms of abundance and richness. As many specialist farmland birds have suffered severe declines since the 1970s (DEFRA 2013), they are typically the target of habitat management improvements on UK farmland. Many of these specialist farmland species are selected for monitoring in Farmer Clusters, where practitioner monitoring is an important activity carried out by these groups (McHugh et al. 2022). Therefore, identifying farmland bird indicator species whose abundance and richness on farmland correlate well with these farmland specialist species is of particular interest. The results presented here will help surveyors understand how individual bird species monitoring relates to measures of community abundance and diversity, so they can understand potential limitations. It is expected that:

1. A similar suite of bird indicator species will be identified on the English and Scottish Farmer Clusters as both study regions are dominated by arable farming and provide broadly comparable landscapes and management regimes for farmland birds (Marston et al. 2023). Furthermore, the same specialist farmland bird species, such as Skylark *Alauda arvensis* and Yellowhammer, are widespread across both countries and consistently respond to variations in farmland management;
2. The strongest indicators of specialist farmland birds (i.e., species reliant on resources provided through traditional farming practices) will be positively related to the species richness and/or abundance of specialist birds only and will be less strongly related to the wider bird community abundance and richness, owing to their heightened dependency on farmland ecosystems (Siriwardena et al. 2000; Siriwardena, Baillie, and Wilson 1998);
3. Other indicator species will be indicative of wider farmland bird abundance and richness;
4. Species demonstrating the strongest relationships with groups of specialist species will be those known to be influenced by farmland habitat management.

## 2 Methods

### 2.1 Monitoring Areas

We studied bird communities across two mixed‐arable Farmer Clusters in the United Kingdom. The Farmer Clusters selected were located in England and Scotland, which have the highest proportions of arable land cover among the four UK nations (31.56% and 7.5% of total land cover, respectively), in contrast to Wales and Northern Ireland where livestock and permanent grassland predominate (Marston et al. 2023). The Cranborne Chase Farmer Cluster, located in the South of England (hereafter English Farmer Cluster), comprised 19 farms between 92 and 1300 ha each and covered a total area of 8400 ha. As part of their landscape‐scale management activities, the English Farmer Cluster implemented a number of actions that may positively impact bird communities, including planting new hedgerows, adding nest boxes for barn owls and swifts and sowing a new network of pollen and nectar flower strips. The second study area was the Buchan Farmer Cluster, located in North‐East Scotland (hereafter Scottish Farmer Cluster), which was made up of eight farms between 2022 and 2024, which are between 80 and 525 ha in size and cover a total area of 1214 ha. Landscape‐scale habitat management actions on the Scottish Farmer Cluster focused on sowing winter bird mixes and wildflower strips, using minimum tillage and cover cropping. The English Farmer Cluster contains a mix of organic and conventional farms, whereas the Scottish Farmer Cluster is entirely conventional in its approach. These were sites established during the FRAMEwork project (2020–2025) (Nichols et al. 2025).

A spatially balanced survey design was implemented to ensure that surveys adequately represented the Farmer Cluster landscape. The English Farmer Cluster was comprised of one continuous landscape and was overlaid with a 1‐km square grid as a basis of a spatially stratified sampling scheme. Any squares that contained land outside the Farmer Cluster, or had less than 75% agricultural land coverage, were excluded from surveys. The Scottish Farmer Cluster was composed of nine non‐adjacent farms, 1‐km square's were therefore overlaid at the farm rather than landscape level; however, one farm was excluded from the current analysis as it was only surveyed in the baseline year. The percentage cover of the four main habitat classes (non‐irrigated arable land, herbaceous area, herbaceous linear, woody area) recorded across the study's survey squares is outlined (Appendix S1). Furthermore, linear models were constructed to test if arable and semi‐natural land cover differed significantly between the two study areas, with results suggesting that no regional differences were found (Appendix S2).

### 2.2 Field Surveys

**FIGURE 1:**
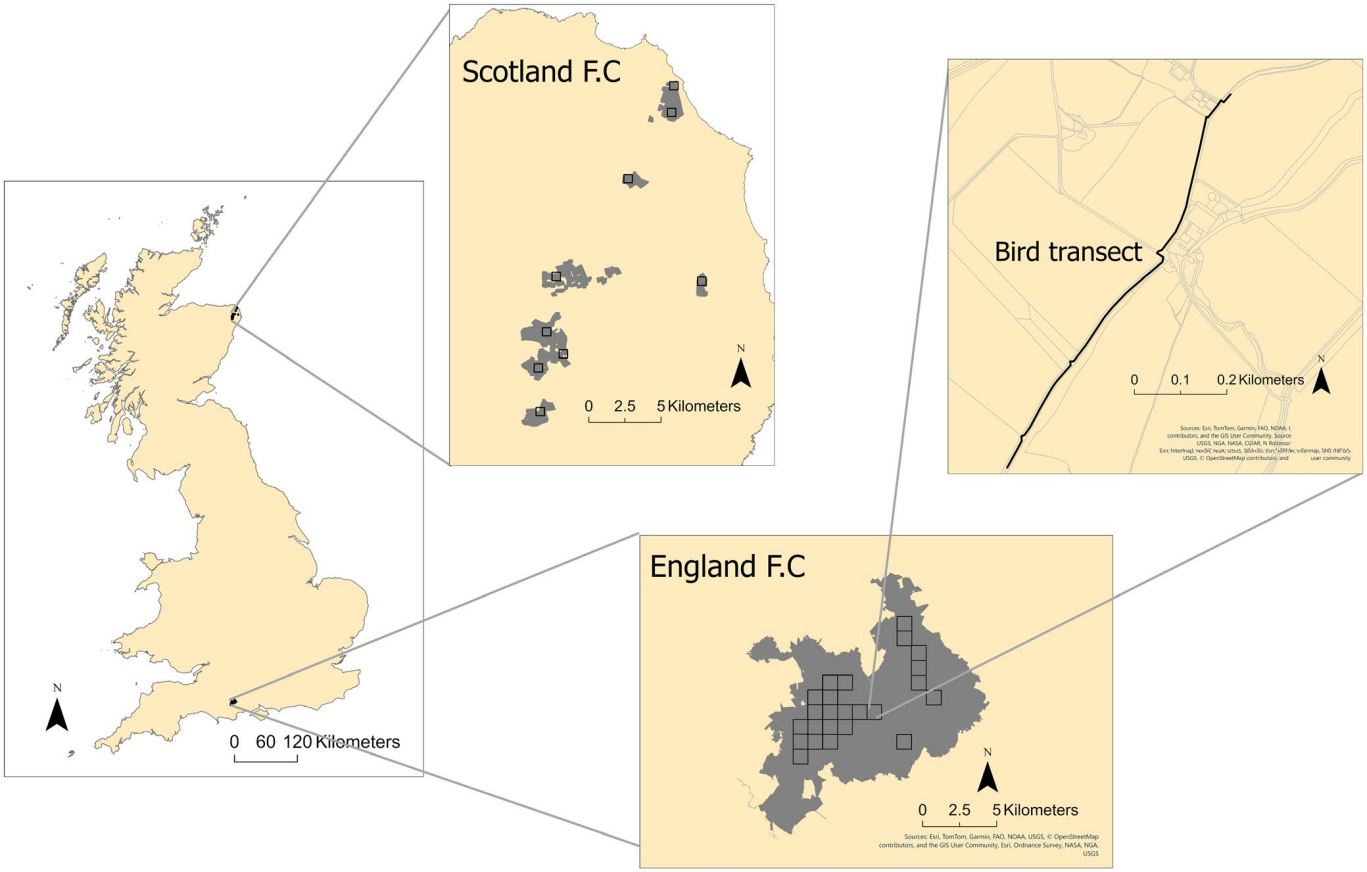
Map of the United Kingdom showing the locations of the Scottish and English Farmer Clusters, the Farmer Cluster configurations and an example bird survey transect.

The FRAMEwork breeding bird survey protocol used a modified version of the British Trust for Ornithology's breeding bird survey (BTO et al. 2018). Within each survey square, a single 1 km‐long transect was established, oriented either north–south or east–west, in a straight line whenever feasible (Figure 1). Ideally, transects were positioned in the middle of each survey square to maintain a separation distance of 1 km, with a minimum distance of 200 m between them, and further divided into 10 100 m segments. The start and end points of each segment were identified in a GIS system, and these points were subsequently uploaded to a handheld GPS device for use in the field. The monitoring activities were carried out twice during the breeding season each year from 2022 to 2024 in Scotland and 2021 to 2024 in England, specifically between 1st of April and 15th of May and a second period from 16th of May to 30th of June (Carrascal and Del Moral 2020).

Transects were walked at least 1 h after sunrise to avoid the period of peak bird activity and were completed by mid‐morning. Multiple transects were often walked in one morning. Transect lines were surveyed at a consistent pace of approximately 5 min per 100 m section. All birds identified visually or acoustically on either side of the transect line were recorded, including adults and juveniles. The recording process categorised birds based on where they were initially seen (estimated distances), including: (1) within 25 m on either side of the transect line; (2) between 25 and 100 m on either side of the line; (3) > 100 m, encompassing birds situated beyond the 1 km square boundary but within the specified distance band; and (4) birds in flight. Distances were measured perpendicular to the transect line.

Surveys were conducted in good weather conditions, and weather was recorded using the following British Trust for Ornithology codes: cloud coverage—0%–33%, 33%–66%, 66%–100%; rain—none, drizzle, showers; wind—calm, light, breezy; and visibility—good, moderate, poor (BTO, JNCC, and RSPB 2018).

### 2.3 Indicator Species Selection

This paper focuses on the role of farmland bird species that have been defined as farmland specialists by Siriwardena, Baillie, Buckland, et al. (1998) and Sharps et al. (2023) as Corn Bunting, Goldfinch *Carduelis carduelis* , Grey Partridge, Lapwing, Linnet *Linaria cannabina* , Skylark, Starling *Sturnus vulgaris* , Stock Dove *Columba oenas* , Tree Sparrow *Passer montanus* , Turtle Dove *Streptopelia turtur* , Whitethroat *Sylvia communis* and Yellowhammer.

### 2.4 Data Analysis

Data analysis was conducted in Rv4.2 (R Core Development Team 2023).

The first set of analyses focused on data collected between 2021 and 2023. To understand if the wider bird communities (i.e., entire suite of birds present on farmland) differed between the Scottish and English Farmer Clusters, we performed community dissimilarity analysis. First, Bray–Curtis dissimilarity (Roberts 2017) was calculated from the wider farmland bird community matrix using the ‘vegan’ package (Oksanen et al. 2025), and a non‐metric multidimensional scaling (NMDS) plot of the two landscapes was created to visualise patterns in species composition. To analyse the community dissimilarity, ‘country’ was tested in a Permutational Multivariate Analysis of Variance (PERMANOVA) using the ‘vegan’ package with 999 permutations. Next, to determine the multivariate spread from the centroid, PERMDISP was performed using the ‘vegan’ package, assessing the effect of ‘country’ using a ‘centroid’ analysis type, which was tested with 999 permutations. Results of both the PERMANOVA and PERMDISP are reported as *F*‐statistics. The results indicated that species composition differed between countries. Subsequent analyses therefore focus on countries separately, as we expect different bird communities to be represented by different indicator species.

Potential co‐linearity between the proposed indicator species was further examined using Pearson's correlation plots to determine if the range of indicator species can be narrowed on the basis of positive correlations between species.

The farmland bird community data were visualised through Farmer Cluster level NMDS, which was again built using Bray–Curtis dissimilarity calculations. The ‘envfit’ function in the ‘vegan’ package was used to identify how bird abundance (individual species, specialist farmland bird abundance and total bird abundance) and richness (specialist farmland bird richness and total bird richness) related to the bird community ordinations. The ‘envfit’ function runs 999 permutations to identify significant relationships, and only significant factors are drawn onto the final figures. Potential correlations identified through this method formed additional hypotheses to test further.

Specialist farmland bird species identified through the aforementioned NMDSs as having potential correlations with measures of abundance and/or richness were selected. To examine the ability of these individual specialist farmland bird species (Table 1) to explain six community‐level measures: total wider bird abundance, total wider bird richness and total wider bird abundance weighted by total wider bird richness, or specialist farmland bird abundance, specialist farmland bird richness and specialist farmland bird abundance weighted by specialist farmland bird richness; a series of generalised linear mixed effects models (GLMMs) were built. At this stage, individual species were included as response variables to identify species that respond to underlying community‐level patterns. Measures of total wider and specialist farmland bird abundance (i.e., our explanatory variables) were specific to each response variable as measures did not include the abundance of the response variable in totals. For example, in the model of Corn Bunting abundance (response variable) and specialist farmland bird abundance (explanatory variable), no Corn Bunting counts were included in the explanatory variable. In the analysis of the English and Scottish bird data, GLMMs included factorised survey round and year nested in survey square as a random effect to control for temporal variability in bird abundances (Bolker et al. 2009). Where issues with model fit occurred (i.e., model singularity or non‐convergence), the model was simplified by including survey round and, if necessary, year as a fixed rather than a nested random effect. Poisson GLMMs were built using the ‘lme4’ package and negative binomial GLMMs with ‘glmmTMB’ (Bates et al. 2015; Brooks et al. 2017). GLMM assumptions were evaluated using the ‘DHARMa’ package (Hartig 2024). Simulated residuals were visually inspected via Q–Q plots, residuals versus predicted values and models were tested for overdispersion and zero inflation. No evidence of overdispersion or zero inflation was detected in any model. A number of models displayed deviations in their quantile tests; in these cases, models were re‐fitted using a negative binomial error structure to account for distributional issues. Model assumptions were therefore considered adequately met.

**TABLE 1:** UK farmland specialists defined by Siriwardena, Baillie, Buckland, et al. (1998).

Farmland specialists	England M ± SE	Scotland M ± SE
Corn Bunting	4.51 ± 0.36	3.56 ± 7.68
Goldfinch	3.96 ± 4.12	7.17 ± 9.62
Grey partridge	0.16 ± 0.69	0.12 ± 0.41
Lapwing	0.13 ± 0.53	0.00 ± 0.00
Linnet	4.39 ± 0.45	14.03 ± 3.79
Skylark	6.73 ± 6.16	18.20 ± 1.96
Starling	0.86 ± 0.21	17.88 ± 4.95
Stock Dove	1.16 ± 0.18	0.24 ± 0.18
Tree sparrow	0.00 ± 0.00	6.62 ± 1.70
Turtle dove	0.00 ± 0.00	0.00 ± 0.00
Whitethroat	1.60 ± 0.16	1.35 ± 2.17
Yellowhammer	2.11 ± 0.23	8.53 ± 1.08

*Note:* The average number of individuals recorded per transect survey round ± the standard error for each Farmer Cluster, from the period 2021 to 2023.

To further test the suitability of the indicator species identified through the preceding set of models, analysis was conducted on the farmland bird data collected from the English and Scottish Farmer Clusters in 2024. Specifically, two GLMMs were built for each Farmer Cluster relating the total bird abundance and specialist bird abundance (response variables) to abundance counts per survey transect for each of the species identified as significant through the previously described GLMM analysis (explanatory variables). GLMMs were either Poisson or negative binomial distributed and contained the nested random effects structure ‘survey round’ within ‘transect section’ within ‘survey square’. Models relating to bird species richness were precluded from analysis due to model convergence issues. GLMM assumptions were again evaluated using the ‘DHARMa’ package (Hartig 2024).

## 3 Results

A total of 192 bird surveys were conducted across 24 1‐km squares in England, with two surveys conducted each year between 2021 and 2023. In Scotland, 48 bird surveys were conducted in total. One‐kilometre squares were surveyed twice per year across nine squares in 2022, eight squares in 2023 and seven squares in 2024.

**FIGURE 2:**
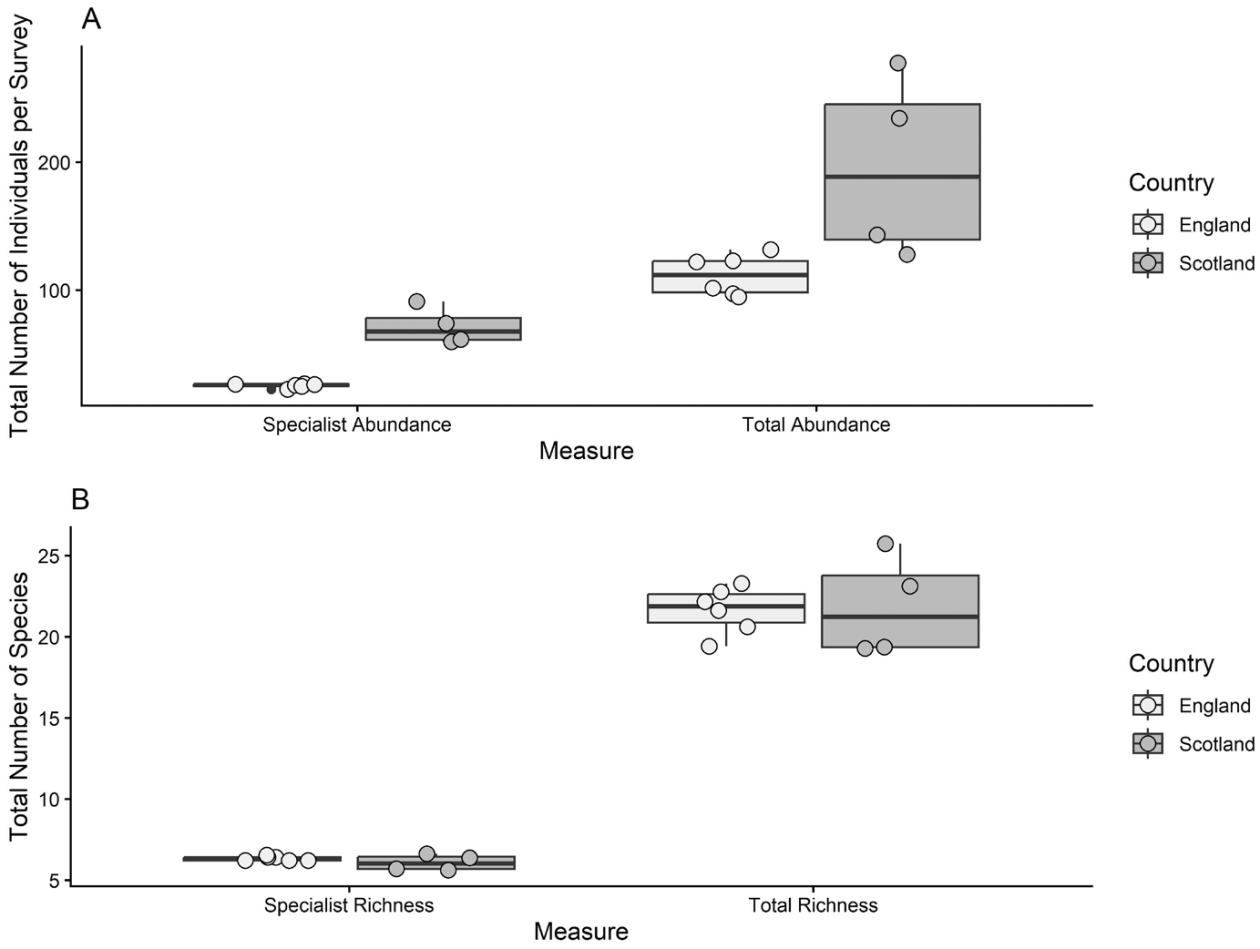
Average total and specialist bird abundance (A) and richness (B) of transects within a given round and year, between 2021 and 2023, showing median (horizontal line), interquartile range (box), solid whisker extending to the upper quartile plus 1.5 times the interquartile range or maximum value if smaller. The scatter depicts average abundance/diversity in each survey round.

**FIGURE 3:**
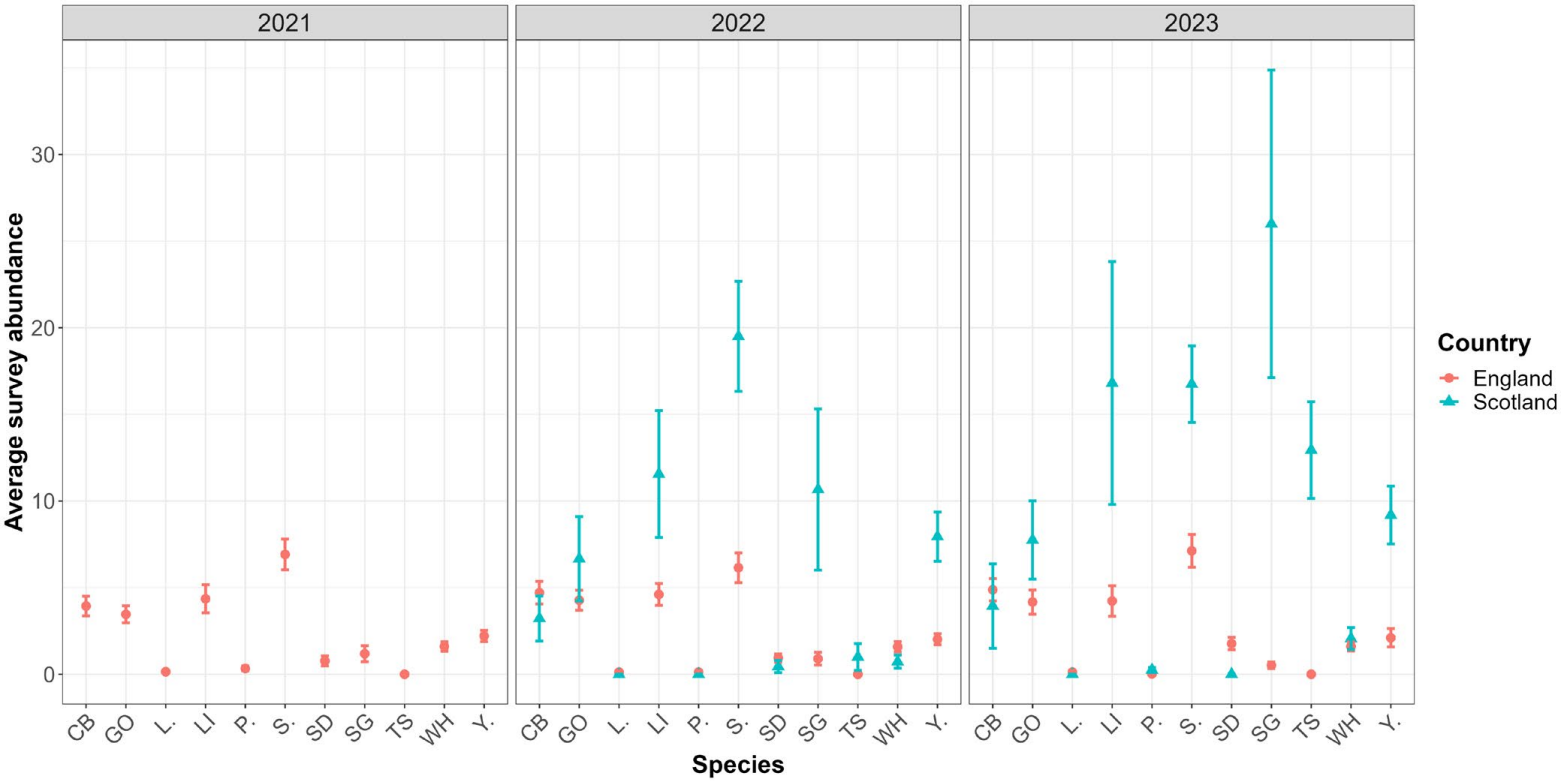
Average abundance of farmland specialist species recorded per survey, per year, ±the standard error for each Farmer Cluster between 2021 and 2023. Turtle Dove were not included as they were absent for both study areas. CB, Corn Bunting; GO, Goldfinch; L., Lapwing; LI, Linnet; P., Grey Partridge; S., Skylark; SD, Stock Dove; SG, Starling; TS, Tree Sparrow; WH, Whitethroat; Y., Yellowhammer.

On the English Farmer Cluster between 2021 and 2023, 16,101 birds were recorded, while 6521 were recorded on the Scottish Farmer Cluster. The English Farmer Cluster was dominated by observations of Woodpigeon *Columba palumbus* (19.51%) and Rook *Corvus frugilegus* (13.35%), while the Scottish Farmer Cluster had a more even distribution of observations, with Skylark (9.45%) and Starling (9.32%) most predominant (Appendix S3). In both Farmer Clusters, just six species made up 50% of the species recorded within each of the Farmer Clusters (Appendix S3). Of all species recorded, 22.9% and 37.1%, respectively, were specialist farmland birds. The average total bird species richness (i.e., the average number of bird species recorded per survey) and specialist farmland bird richness (i.e., the average number of specialist farmland bird species) recorded per survey was, however, similar between England (total = 21.65 ± 0.48; specialist = 6.33 ± 0.11) and Scotland (total = 21.72 ± 1.24; specialist = 6.06 ± 0.30; Figure 2). Specialist farmland bird communities were more even in England compared to Scotland, and the abundance of specialist farmland bird species varied considerably between the surveyed Farmer Clusters across and between years (Figure 3; Table 1). Further descriptive statistics can be found in Appendix S4.

**FIGURE 4:**
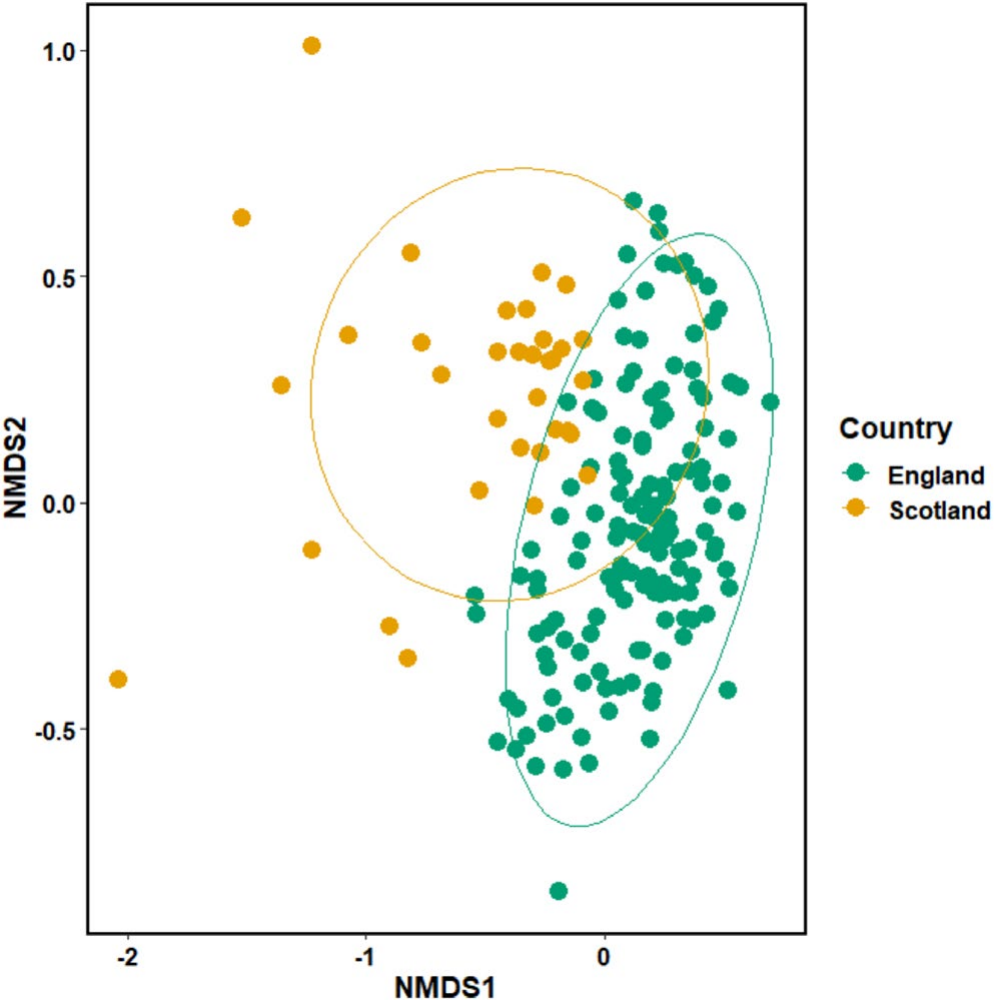
NMDS of farmland bird survey data. Each point on the plot represents the farmland bird community recorded during one bird transect survey. Points closer together represent communities that are more similar in their composition. Stress value = 0.20.

Both English and Scottish Farmer Clusters appear to have distinct bird communities as shown in an NMDS analysis (Figure 4; PERMANOVA: *F*_1,183_ = 22.396, *p* = 0.001). The analysis of dispersion suggested this was not caused by variation within the Farmer Clusters, but between them (PERMDISP: *F*_1,182_ = 2.2354, *p* = 0.143). We would therefore expect that the communities may be best explained by different indicator species; because of this, the English and Scottish bird data were treated separately in subsequent analysis.

The Pearson's correlation plot of potential English indicator bird species (Table 1) revealed that correlation in abundance was generally low between species pairs; the highest correlation seen was a moderate value of 0.50 between Corn Bunting and Skylark (Appendix S5). The Pearson's correlation plot generated with the Scottish bird species again showed that correlations were generally low between species pairs, but a high correlation value of 0.92 was noted between Linnet and Corn Bunting and a moderate value of 0.51 between Linnet and Goldfinch (Appendix S6).

**FIGURE 5:**
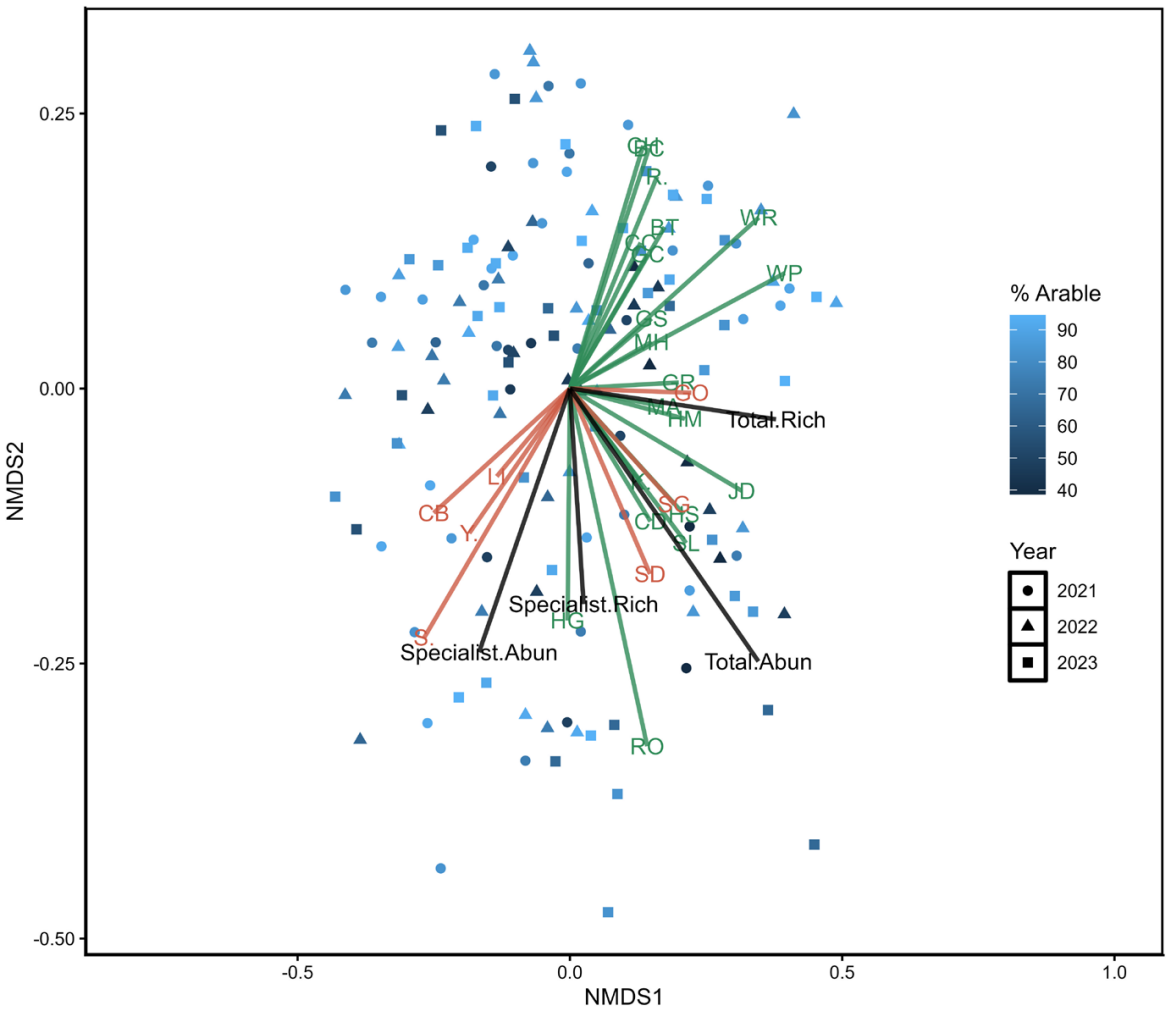
NMDS community analysis of farmland bird survey data from the English Farmer Cluster using Bray–Curtis dissimilarity distances of bird species (specialists = orange, generalists = green), specialist abundance, specialist richness, total abundance and total richness, stress value = 0.22. Each point represents the farmland bird community seen during a bird survey, coloured according to the percentage arable land cover on the 1‐km^2^ survey square. The bird species depicted on the NMDS plot are BC, Blackcap; BT, Blue Tit; CB, Corn Bunting; CC, Chiffchaff; CD, Collared Dove; CH, Chaffinch; GC, Goldcrest; GO, Goldfinch; GR, Greenfinch; GS, Great Spotted Woodpecker; HG, Herring Gull; HM, House Martin; HS, House Sparrow; JD, Jackdaw; K., Kestrel; LI, Linnet; MA, Mallard; MH, Moorhen; R., Robin; RO, Rook; S., Skylark; SD, Stock Dove; SG, Starling; SL, Swallow; WP, Woodpigeon; WR, Wren; Y., Yellowhammer.

**FIGURE 6:**
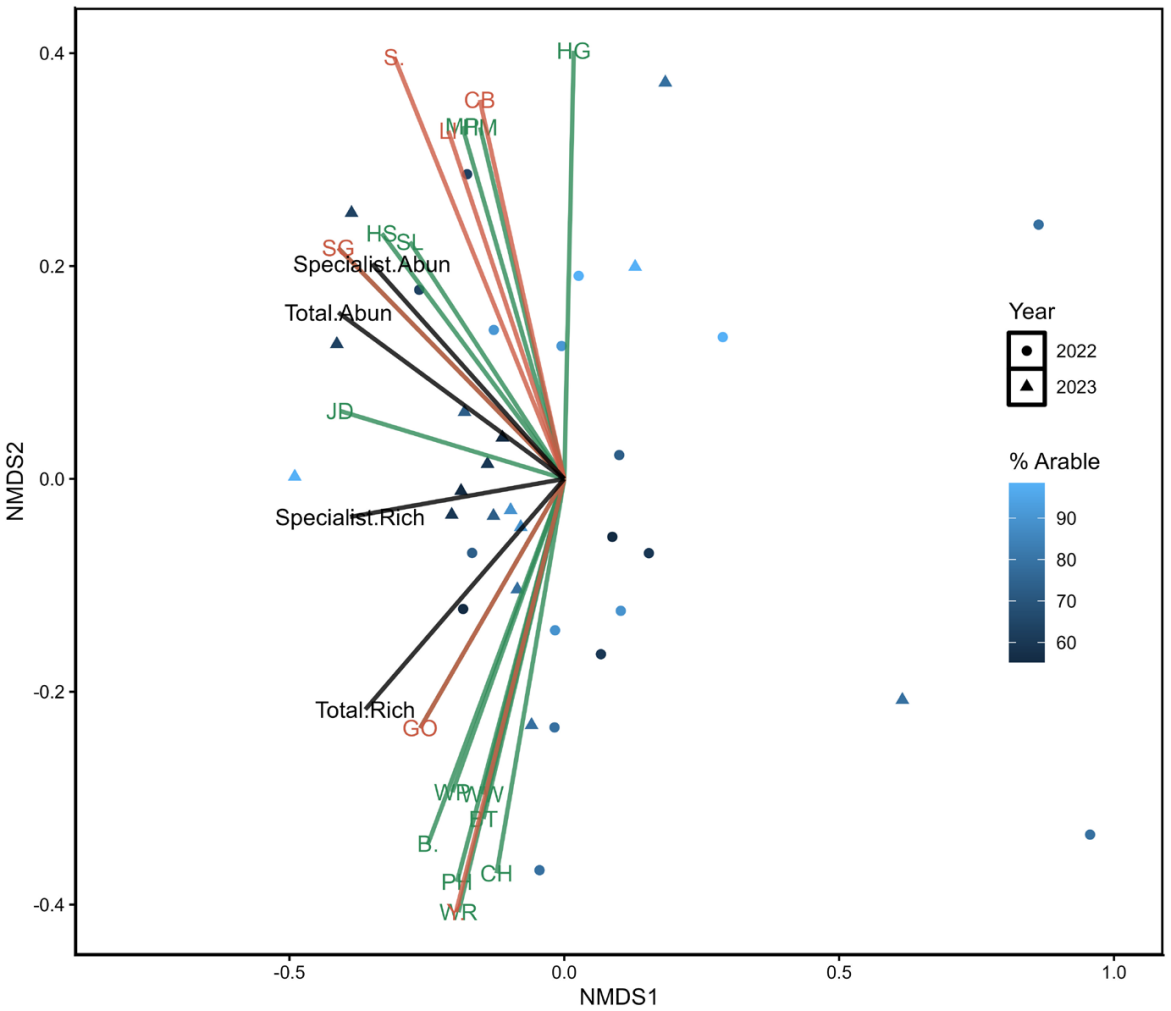
NMDS community analysis of farmland bird survey data from the Scottish Farmer Cluster using Bray–Curtis dissimilarity distances of bird species (specialists = orange, generalists = green), specialist abundance, specialist richness, total abundance and total richness, stress value = 0.15. Each point represents the farmland bird community seen during a bird survey, coloured according to the percentage arable land cover on the 1‐km^2^ survey square. The bird species depicted on the NMDS plot are B., Blackbird; BT, Bluetit; CB, Corn Bunting; CG, Canada Goose; GO, Goldfinch; HG, Herring Gull; HM, House Martin; HS, House Sparrow; JD, Jackdaw; LI, Linnet; MP, Meadow Pipit; PH, Pheasant; S., Skylark; SG, Starling; SL, Swallow; WP, Woodpigeon; WR, Wren; WW, Willow warbler; Y., Yellowhammer.

Further NMDS analysis of Farmer Cluster level data showed seven of the 12 potential specialist farmland bird indicator species were indicative of community‐level measures in England and six in Scotland (Figures 5 and 6). These specialist farmland bird indicator species could be divided into three categories: those representing specialist farmland bird abundance, those more indicative of the wider bird communities present on farmland and species that ordinated differently in both countries. In England and Scotland, Corn Bunting (CB), Linnet (Li) and Skylark (S) were most strongly ordinated with specialist farmland bird abundance and opposed measures of total species richness. Yellowhammer (Y) and Starling (SG) demonstrated different relationships in opposing Farmer Clusters. Yellowhammer ordinated alongside specialist farmland bird species abundance in England, while Starling ordinated alongside these species in Scotland. In both countries, Goldfinch (GO) ordinated closely with total bird community species richness. These findings provided hypotheses to test further with GLMM analysis, splitting potential indicator species between those ordinating with specialist farmland bird community measures and total bird community measures. Yellowhammer and Starling were excluded as potential indicators at this point owing to their differential ordination in Scotland and England. The value of Stock Dove as a wider bird community indicator was investigated in England, despite not being highlighted in the Scottish data as it was infrequently recorded in Scotland.

Analysis of the English farmland bird data showed that the potential specialist farmland bird indicators Skylark, Corn Bunting and Linnet correlated to varying degrees with the measures of specialist farmland bird abundance and richness. Only Linnet positively correlated to all the specialist farmland bird abundance and richness measures, but Skylark abundance was positively related to two of the three measures (Table 2). Corn Bunting abundance was positively associated with the weighted specialist abundance metric (Table 2). This suggests that Corn Buntings occur in higher numbers in areas where specialist farmland species are not only present but also relatively abundant, indicating that their occurrence reflects both richness and abundance within the wider specialist farmland bird community.

**TABLE 2:** Summary of relationships between selected potential specialist farmland bird indicator species and total specialist bird abundance, specialist richness and specialist bird abundance weighted by specialist bird richness using the English Farmer Cluster data.

Species	Total specialist bird abundance	Specialist richness	Specialist bird abundance weighted by specialist bird richness
Corn Bunting	N.S.	N.S.	+***
Linnet	+*	+**	+***
Skylark	+*	N.S.	+***

*Note:* Relationships were examined using generalised linear mixed effects models. Full model output is provided in Appendix S7. + = positive correlation; significance is recorded as: *p = 0.05; **p = 0.01; ***p = 0.001; N.S., not significant.

Stock Dove and Goldfinch were considered as indicators of wider farmland bird communities on the English Farmer Cluster due to their ordinations on the NMDS (Figure 5). GLMM analysis showed that Stock Dove and Goldfinch were both strongly positively correlated to the three measures of total bird abundance and richness (Table 3).

**TABLE 3:** Summary of correlations between selected potential wider farmland bird community indicator species and total bird abundance, total richness and total bird abundance weighted by total bird richness using the English Farmer Cluster data.

Species	Total bird abundance	Total richness	Total bird abundance weighted by total bird richness
Stock Dove	+***	+***	+***
Goldfinch	+***	+***	+***

*Note:* Relationships were examined using generalised linear mixed effects models. The full model output is provided in Appendix S8. +, positive correlation; significance is recorded as: *p = 0.05; **p = 0.01; ***p = 0.001; N.S., not significant.

Analysis of the Scottish farmland bird data showed that Linnet was the most consistent indicator for farmland specialists and their abundance was found to positively associate with all three specialist farmland bird measures. Corn Buntings were also highlighted as a potential specialist farmland bird indicator species as they were positively correlated with specialist farmland bird abundance and specialist farmland bird abundance weighted by specialist farmland bird richness (Table 4).

**TABLE 4:** Summary of relationships between selected potential specialist farmland bird indicator species and total specialist bird abundance, specialist richness and specialist bird abundance weighted by specialist bird richness using the Scottish Farmer Cluster data.

Species	Total specialist bird abundance	Specialist richness	Specialist bird abundance weighted by specialist bird richness
Corn Bunting	+**	N.S.	+**
Linnet	+***	+***	+***
Skylark	N.S.	N.S.	N.S.

*Note:* Relationships were examined using generalised linear mixed effects model. The full model output is provided in Appendix S9. +, positive correlation; significance is recorded as: *p = 0.05; **p = 0.01; ***p = 0.001; N.S., not significant.

Goldfinch was identified as a potential wider bird community indicator species in the Scottish NMDS (Figure 6). Further statistical analysis of their relationships with the wider bird community showed that Goldfinch were the most likely indicator species as their abundance was positively correlated with the three measures of bird abundance and richness that were investigated (Table 5).

**TABLE 5:** Summary of relationships between selected potential wider farmland bird community indicator species and total bird abundance, total richness and total bird abundance weighted by total bird richness using the Scottish Farmer Cluster data.

Species	Total bird abundance	Total richness	Total bird abundance weighted by total bird richness
Goldfinch	+**	+*	+**

*Note:* Relationships were examined using generalised linear mixed effects models. The full model output is provided in Appendix S10. +, positive correlation; significance is recorded as: *p = 0.05; **p = 0.01; ***p = 0.001; N.S., not significant.

Further analysis focused on the 2024 bird data, which was used to verify the indicators identified during the 2021–2023 period. In England, all proposed indicator species again showed significant relationships with specialist or total bird abundance (Table 6). In Scotland, Corn Bunting and Linnet showed significant positive relationships with specialist farmland bird abundance, though Goldfinch was not significantly positively related to total bird abundance, but a positive trend was recorded (Table 6).

**TABLE 6:** Poisson or negative binomial generalised linear mixed effects models for (A) England and (B) Scotland, with a log link, of (1) total specialist bird abundance and (2) total bird abundance in relation to potential indicator species identified in Tables 2 and 3.

Country	Response variable	Fixed effects	Estimate ± SE	*Z*	*p*
(A) England	(1) Specialist abundance (NB)	Intercept	0.52 ± 0.06	8.24	**< 0.001**
		Corn Bunting	0.08 ± 0.01	6.77	**< 0.001**
		Linnet	0.18 ± 0.02	10.07	**< 0.001**
		Skylark	0.23 ± 0.02	10.24	**< 0.001**
	(2) Total bird abundance (NB)	Intercept	2.24 ± 0.08	26.83	**< 0.001**
		Goldfinch	0.12 ± 0.03	3.94	**< 0.001**
		Stock Dove	0.15 ± 0.06	2.63	**< 0.01**
(B) Scotland	(1) Specialist abundance (NB)	Intercept	1.53 ± 0.14	11.11	**< 0.001**
		Corn Bunting	0.27 ± 0.10	2.75	**< 0.01**
		Linnet	0.06 ± 0.02	4.01	**< 0.001**
	(2) Total bird abundance (P)	Intercept	3.03 ± 0.10	29.26	**< 0.001**
		Goldfinch	0.08 ± 0.05	1.67	0.10

*Note:* Significant values are in bold. The family used in models is indicated by P (Poisson) or NB (negative binomial).

## 4 Discussion

Indicator species can act as a practical tool for assessing the health of broader wildlife communities, and the results presented here aim to improve our understanding of farmland bird indicator species in the United Kingdom, highlighting the potential limitations of biodiversity monitoring with indicator species, as well as supporting informed decision‐making on farmland. The study evaluations revealed some consistency between specialist farmland birds and total bird community measures, and individual bird species in two mixed farmland landscapes. Of the 12 specialist farmland species investigated, seven were found to have potential associations with bird community data in England and in Scotland. However, species‐specific relationships with community measures were not always consistent (e.g., Yellowhammer and Starling). The study results also support the context‐specific use of Corn Bunting, Linnet and Skylark in estimating specialist farmland bird community abundance, and Goldfinch and Stock Dove in estimating overall community abundance.

A species' distribution represents a gradient, ranging from optimal habitat to tolerable habitat. As a species' total abundance increases, the likelihood of it occurring in less suitable habitats increases. For species classified as specialist farmland birds, their suitability as an indicator species for wider bird diversity or specialist farmland birds appears to relate, in part, to their abundance. This is consistent with the case of Goldfinch and Yellowhammer. Goldfinches are undergoing national population increases (Heywood et al. 2024), and only weakly corresponded with the distribution of other specialist farmland species in both England and Scotland, but are highly correlated to total species richness. Similarly, Yellowhammers in Scotland, where they are more abundant (Heywood et al. 2024), correlated less strongly with other specialist farmland species, compared to in England where they are less abundance.

The results highlight the importance of considering specific conservation goals when selecting farmland bird indicator species, for example, the promotion of general bird diversity or specialist declining species on farmland. The latter group was represented by Corn Bunting, Linnet and Skylark and their abundance was highly correlated to one another in both Scottish and English landscapes. They represent a group of ‘true arable specialists’, they are closely linked to specialist farmland bird abundance and, in England, opposed total community richness and abundance measures. These specialist species also represent a range of habitat preferences and dietary niches within farmland ecosystems. Corn Buntings and Skylarks nest in open fields, adults feed on seeds, but the diet of nestlings almost exclusively comprises invertebrates (Holland et al. 2012). In contrast, Linnets nest along field boundaries and are specialist seed eaters at all life cycle stages (Holland et al. 2006). The diversity of this group, therefore, allows them to collectively provide a comprehensive picture of the health of specialist farmland bird species. These species are relatively easy to monitor due to their distinctive calls or breeding displays, making them practical for large‐scale surveys by novices and long‐term monitoring programmes. The Goldfinch is similarly recognisable and was linked to total bird species richness in both Farmer Clusters between 2021 and 2023.

Specialist farmland bird species which were either absent from surveys or only present in very low numbers should not be discounted as indicator species in other landscapes. Lapwing, for example, were absent in Scotland and only present in very low numbers in England. This species is highly sensitive to changes in agricultural practices and land use (Taylor and Grant 2004; Plard et al. 2020) and has declined by 62% in England since 1967 (Heywood et al. 2024); it is now Red listed under the Birds of Conservation Concern 5 (Stanbury et al. 2021). When a species population has dropped below a critical value, its role as an indicator of the wider reference community may be reduced. It is therefore likely that the results presented here are region specific and should therefore be repeated in different landscape and farming contexts to generate information on regionally specific indicator species for monitoring.

For other specialist farmland bird species considered, a lack of associations, and hence indicator potential, can be linked to the survey methods employed. Grey Partridges, for example, are normally surveyed using a species‐specific method, for example, 2 h dusk and dawn Spring and Autumn Grey Partridge counts are conducted to estimate their abundance (Ewald et al. 2009), a significantly different method to the one used here. The Grey Partridge is regularly cited as an indicator of sustainable agriculture (Beier and Jánoska 2023), but not of biodiversity. Studies have shown that factors such as pesticide use, loss of hedgerows and monoculture cropping can significantly affect Grey Partridge populations (Boatman et al. 2004). We recommend that further research be conducted to verify the relationship between Grey Partridge and other easily surveyed bird species or farmland biodiversity metrics.

While this study demonstrates the potential of indicator bird species for streamlined monitoring, some limitations should be acknowledged. Sample sizes were smaller in Scotland compared to England, which may reduce the robustness of regional comparisons and limit the generalisability of results across the wider United Kingdom. Variation in species detectability also introduces potential bias into our results, despite consistent protocols being applied. Future work should therefore calculate and integrate detectability corrections for cryptic species and strive for a more balanced sampling effort across study areas. Despite this, there is potential to build on this farmland bird indicators work and develop multi‐taxa monitoring schemes that align with broader agri‐environment objectives, for example, by linking bird‐based indicators with those for pollinators, plants or soil. This would provide a more holistic evidence base to evaluate the ecological and agricultural benefits of AES and ensure that management actions deliver for biodiversity at multiple trophic levels.

The methods and results presented in this study could be used as a first step in designing a citizen science‐based farmland bird monitoring programme for Farmer Clusters. Stratified sampling of a narrow range of farmland bird species across Farmer Clusters using protocols that are simple and easy to apply should increase uptake and participation by farmers (Ebitu et al. 2021). Repeating the analysis conducted here in different regions and farming contexts will also ensure that the indicator species monitoring conducted is relevant to farmers' needs. Following this additional analysis, protocols should be trialled by farmers and other citizen scientists, and recorder feedback sought (e.g., Garratt et al. 2019) to determine how willing and able farmers are to participate in monitoring. Furthermore, although this study did not assess the functional roles of indicator species, linking them to their ecosystem service contributions would enhance the value of such monitoring by highlighting both their ecological significance and their potential benefits for sustainable farming systems. Such insights would also be relevant in contexts where professional monitoring is required to support result‐based agri‐environment scheme payments or to measure biodiversity credits. Using relevant indicator species in place of complete community surveys not only reduces data collection and processing demands but also provides policymakers with a stronger evidence base for valuing and targeting AES payments by demonstrating not only the biodiversity benefits but also the wider ecological and economic services supported by farmland bird communities.

## Author Contributions

**N. M. McHugh:** conceptualization (lead), formal analysis (lead), funding acquisition (supporting), investigation (lead), methodology (lead), writing – original draft (lead). **E. R. Ness:** data curation (supporting), investigation (lead), writing – review and editing (supporting). **R. Nichols:** formal analysis (supporting), methodology (supporting), writing – original draft (supporting), writing – review and editing (supporting). **G. Banks:** data curation (supporting), investigation (supporting), writing – review and editing (supporting). **D. Strong:** data curation (supporting), investigation (lead), writing – review and editing (supporting). **M. W. Young:** data curation (lead), writing – review and editing (supporting). **A. Zuta:** visualization (supporting), writing – review and editing (supporting). **G. S. Begg:** conceptualization (supporting), funding acquisition (lead), methodology (supporting), writing – original draft (supporting), writing – review and editing (supporting).

## Conflicts of Interest

The authors declare no conflicts of interest.

## Supporting information


**Appendix S1:** ece372380‐sup‐0001‐Supinfo.zip.

## Data Availability

All the required data are uploaded as Supporting Information.
