# Supplementary material for: Identification of Farmland Bird Indicator Species for Practitioner Monitoring in the United Kingdom
Source: Ecol Evol. 2025 Nov 16;15(11):e72380. doi: 10.1002/ece3.72380 (PMC12620028; doi:10.1002/ece3.72380)
Supplement: Supplementary file 1 — Appendix S1: ece372380‐sup‐0001‐Supinfo.zip. [file ECE3-15-e72380-s001.zip › ece372380-sup-0001-Supinfo1@10.09.25 Supplementary material_revised.docx]

**Appendix 1**

Percentage cover of non-irrigated arable land, herbaceous areal (natural grassland, inland marshes and modified grassland) and herbaceous linear (grass banks and arable field margins) and woody areal (woodland and transitional scrub) habitats across the studies 32 survey squares.

| **Country** | **Survey square** | **Non-irrigated arable land** | **Herbaceous Areal** | **Herbaceous Linear** | **Woody Areal** |
| --- | --- | --- | --- | --- | --- |
| Scotland | 1 | 72.98% | 23.00% | 1.30% | 0.06% |
| Scotland | 2 | 89.45% | 7.69% | 0.76% | 0.35% |
| Scotland | 3 | 78.66% | 17.49% | 1.07% | 0.12% |
| Scotland | 4 | 98.47% | 0.55% | 0.07% | 0.12% |
| Scotland | 5 | 55.22% | 39.49% | 2.10% | 0.83% |
| Scotland | 6 | 63.44% | 27.27% | 3.27% | 0.41% |
| Scotland | 7 | 78.66% | 17.49% | 1.07% | 0.12% |
| Scotland | 8 | 59.71% | 34.74% | 2.25% | 1.10% |
| England | 9 | 91.43% | 2.86% | 1.31% | 0.77% |
| England | 10 | 45.55% | 49.22% | 0.93% | 1.41% |
| England | 11 | 91.60% | 0.00% | 5.36% | 0.30% |
| England | 12 | 65.98% | 21.55% | 2.55% | 1.39% |
| England | 13 | 56.04% | 12.71% | 1.96% | 20.95% |
| England | 14 | 85.87% | 4.77% | 1.20% | 4.52% |
| England | 15 | 83.22% | 0.00% | 3.06% | 10.64% |
| England | 16 | 94.60% | 0.10% | 1.48% | 1.07% |
| England | 17 | 92.98% | 0.00% | 0.54% | 4.18% |
| England | 18 | 65.11% | 13.53% | 3.22% | 12.40% |
| England | 19 | 90.48% | 6.31% | 0.70% | 0.82% |
| England | 20 | 85.10% | 0.11% | 0.15% | 7.78% |
| England | 21 | 85.36% | 3.47% | 3.12% | 4.10% |
| England | 22 | 73.79% | 13.45% | 2.19% | 2.56% |
| England | 23 | 83.06% | 3.94% | 3.70% | 4.82% |
| England | 24 | 92.09% | 3.09% | 0.28% | 1.11% |
| England | 25 | 50.68% | 34.15% | 1.19% | 4.85% |
| England | 26 | 74.44% | 6.82% | 0.65% | 13.71% |
| England | 27 | 84.96% | 0.00% | 0.72% | 10.31% |
| England | 28 | 72.00% | 9.70% | 2.10% | 13.72% |
| England | 29 | 38.68% | 50.43% | 1.90% | 1.27% |
| England | 30 | 88.16% | 0.52% | 1.58% | 8.11% |
| England | 31 | 48.38% | 26.23% | 3.17% | 16.99% |
| England | 32 | 82.71% | 13.47% | 1.30% | 0.02% |

**Appendix 2**

Linear models comparing (1) Arable land cover and (2) Semi-Natural habitat cover on the English and Scottish Farmer Clusters. Significant values are in bold.

|  | **Fixed effects** | **Estimate ± SE** | **Z-value** | **P** |
| --- | --- | --- | --- | --- |
| **Arable land cover** | Intercept (England) | 0.19 ± 0.03 | 6.26 | **<0.001** |
|  | Scotland | 0.03 ± 0.06 | 0.53 | 0.60 |
| **Semi-natural habitat** | Intercept (England) | 0.56 ± 0.02 | 28.15 | **<0.001** |
|  | Scotland | -0.01 ± 0.04 | -0.15 | 0.88 |

**Appendix 3**

The top 10 percentage contributions of species observations made combined for all surveys from 2021 – 2023 in the Scottish Farmer Cluster (Scotland) and the English Farmer Cluster (England).

| Scotland | | England | |
| --- | --- | --- | --- |
| Species | Percentage | Species | Percentage |
| Skylark | 9.49 | Woodpigeon | 19.51 |
| Starling | 9.32 | Rook | 13.35 |
| Rook | 8.96 | Skylark | 6.02 |
| Carrion Crow | 8.57 | Corn Bunting | 4.03 |
| Linnet | 7.31 | Blackbird | 3.99 |
| House Sparrow | 6.55 | Linnet | 3.93 |
| Jackdaw | 5.83 | Jackdaw | 3.74 |
| Woodpigeon | 4.77 | Goldfinch | 3.55 |
| Yellowhammer | 4.45 | Chaffinch | 3.43 |
| Goldfinch | 3.74 | Wren | 3.01 |

**Appendix 4**

The average total bird abundance (i.e. the average total number of farmland birds recorded per survey) and specialist farmland bird abundance (i.e. the average total number of farmland bird specialists recorded per survey) recorded per survey was higher in Scotland (total = *M ± SE*; total = 191.79 ± 19.38; specialist = 71.09 ± 9.31) compared to England (total = 111.82 ± 5.24; specialist = 25.63 ± 1.10; Figure 1). The total richness of the bird community was higher on the English Farmer Cluster with 90 species being recorded, compared to the 61 species recorded on the Scottish Farmer Cluster across the 2021 – 2023 surveys.

All 24 1-km squares were resurveyed on the English Farmer Cluster in 2024 and analysed separately to the 2021 – 2023 data to confirm the validity of selected indicator species. In 2024 total bird species richness on the English Farmer Cluster was 82. In the 2024 English breeding bird surveys the average total bird abundance was higher than in the proceeding period (total = 139.06 ± 10.47) and specialist farmland bird abundance was comparatively lower (specialist = 17.29 ± 0.87). Seven of the Scottish Farmer Cluster survey squares were resurveyed in 2024. Total bird species richness on the Scottish Farmer Cluster was 57 in 2024, average total bird abundance was 314.56± 13.16 and specialist farmland bird abundance was 80.94 ± 13.15.

The mean abundance per survey of Corn Bunting, Grey Partridge and Whitethroat were similar between England and Scotland, but on average Linnet, Skylark, Starling and Yellowhammer were more abundant in Scotland compared to England (Table 1). Lapwing were present in low numbers on the English Farmer Cluster and absent in Scotland, the opposite was true for Tree Sparrow (Table 1).

**Appendix 5 – Pearsons England**


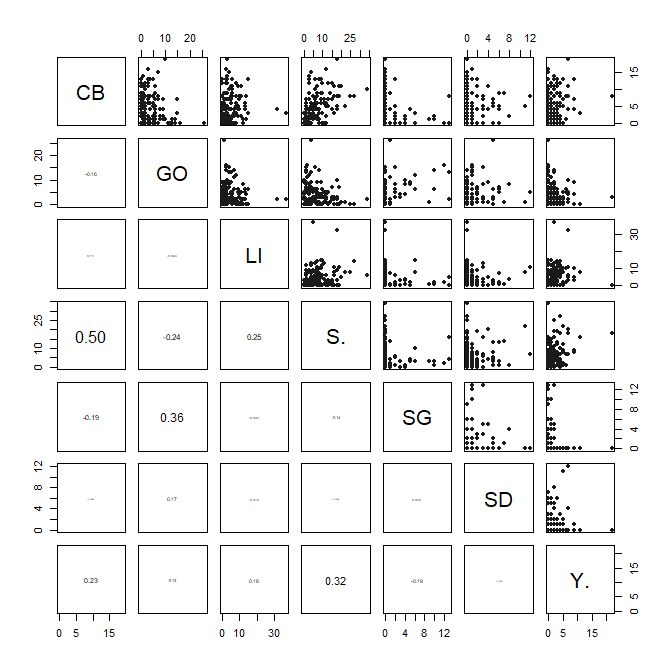


Figure A2. Collinearity plots between the seven proposed farmland bird indicator species for England. Pearson’s correlation values are provided in the lower panels, larger text indicates that variables are more correlated, medium correlation is represented by values greater than 0.5 or less than -0.5 and high correlation, are values greater than 0.7 or less than -0.7. Bird species names are abbreviated; CB = Corn Bunting, GO = Goldfinch, LI = Linnet, S. = Skylark, SG = Starling, SD = Stock Dove, Y. = Yellowhammer.

**Appendix 6– Pearsons Scotland**


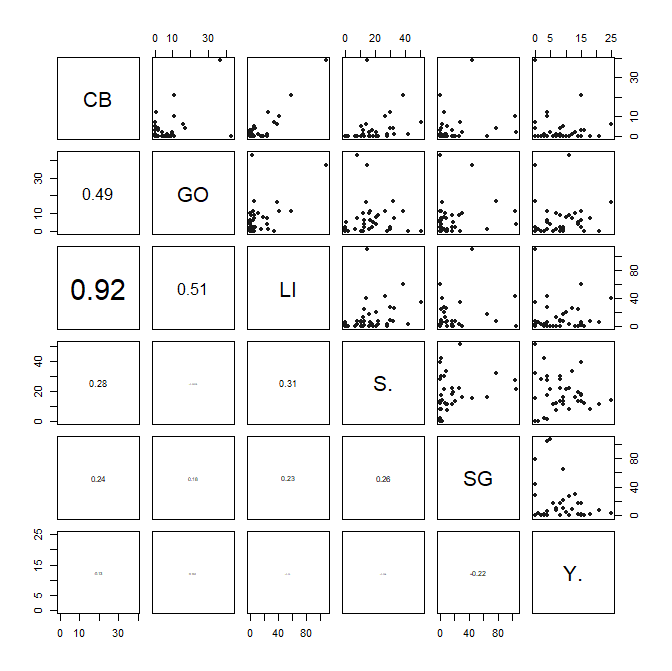


Figure A3. Collinearity plots between the seven proposed farmland bird indicator species for Scotland. Pearson’s correlation values are provided in the lower panels, larger text indicates that variables are more correlated, medium correlation is represented by values greater than 0.5 or less than -0.5 and high correlation, are values greater than 0.7 or less than -0.7. Bird species names are abbreviated; CB = Corn Bunting, GO = Goldfinch, LI = Linnet, S. = Skylark, SG = Starling, Y. = Yellowhammer.

**Appendix 7**

Poisson or Negative Binomial generalised linear mixed effects models, with a log link, of (1) Skylark, (2) Corn Bunting and (3) Linnet abundance on the English Farmer Cluster versus (A) total specialist bird abundance, (B) specialist bird species richness, and (C) specialist bird abundance weighted by specialist bird species richness (richness weighted). Significant values are in bold. The family used in models is indicated by P (Poisson) or NB (Negative binomial).

|  | **Fixed effects** | **Estimate ± SE** | **Z-value** | **P** |
| --- | --- | --- | --- | --- |
| **1.A Skylark (NB)** | Intercept | 0.81 ± 0.37 | 2.20 | **<0.05** |
|  | Log (Specialists) | 0.25 ± 0.11 | 2.23 | **<0.05** |
| **1.B (P)** | Intercept | 1.24 ± 0.33 | 3.87 | **<0.001** |
|  | Specialist richness | 0.04 ± 0.04 | 1.05 | 0.29 |
| **1.C (NB)** | Intercept | -1.19 ± 0.52 | -2.30 | **<0.05** |
|  | Log (richness weighted) | 0.55 ± 0.10 | 5.65 | **<0.001** |
|  | Round (2) | -0.00 ± 0.06 | -0.08 | 0.94 |
| **2. A Corn Bunting (P)** | Intercept | 0.44 ± 0.49 | 0.90 | 0.38 |
|  | Log (Specialists) | 0.06 ± 0.13 | 0.43 | 0.67 |
|  | Year (2022) | 0.17 ± 0.10 | 1.78 | 0.08 |
|  | Year (2023) | 0.21 ± 0.10 | 2.20 | **<0.05** |
|  | Round (2) | 0.34 ± 0.08 | 4.17 | **<0.001** |
| **2.B (P)** | Intercept | 0.10 ± 0.39 | 0.27 | 0.79 |
|  | Specialist richness | 0.08 ± 0.04 | 1.89 | 0.06 |
|  | Year (2022) | 0.18 ± 0.10 | 1.86 | 0.06 |
|  | Year (2023) | 0.22 ± 0.10 | 2.21 | **<0.05** |
|  | Round (2) | 0.35 ± 0.08 | 4.37 | **<0.001** |
| **2.C (NB)** | Intercept | -1.53 ± 0.64 | -2.41 | **<0.05** |
|  | Log (richness weighted) | 0.46 ± 0.12 | 3.93 | **<0.001** |
|  | Round (2) | 0.38 ± 0.08 | 4.66 | **<0.001** |
| **3.A Linnet (P)** | Intercept | -0.34 ± 0.68 | -0.50 | 0.61 |
|  | Log (Specialists) | 0.43 ± 0.22 | 2.00 | **<0.05** |
| **3.B (P)** | Intercept | 0.34 ± 0.37 | 0.92 | 0.36 |
|  | Richness | 0.16 ± 0.05 | 3.07 | **<0.01** |
|  | Round (2) | -0.56 ± 0.08 | -6.89 | **<0.001** |
| **3.C (NB)** | Intercept | -4.29 ± 0.78 | 0.78 | <**0.001** |
|  | Log (richness weighted) | 1.09 ± 0.15 | 7.22 | **<0.001** |

**Appendix 8 -England – total bird community**

Poisson or Negative binomial generalised linear mixed effects models, with a log link, of (1) Goldfinch, (2) Stock Dove abundance on the English Farmer Cluster versus (A) total bird abundance, (B) total bird species richness and (C) total bird abundance weighted by total bird species richness (richness weighted). Significant values are in bold. The family used in models is indicated by P (Poisson) or NB (Negative binomial).

|  | **Fixed effects** | **Estimate ± SE** | **Z-value** | **P** |
| --- | --- | --- | --- | --- |
| **1.A Goldfinch (NB)** | Intercept | -2.15 ± 0.93 | -2.20 | **<0.05** |
|  | Log (Total abundance) | 0.71 ± 0.20 | 3.55 | **<0.001** |
| **1.B (P)** | Intercept | -1.18 ± 0.35 | -3.32 | **<0.001** |
|  | Richness | 0.10 ± 0.01 | 7.20 | **<0.001** |
|  | Round (2) | -0.04 ± 0.09 | -0.47 | 0.64 |
| **1.C (P)** | Intercept | -4.11 ± 1.03 | -3.98 | **<0.001** |
|  | Log (richness weighted) | 0.67 ± 0.13 | 5.05 | **<0.001** |
| **2.A Stock Dove (P)** | Intercept | -8.76 ± 1.85 | -4.72 | **<0.001** |
|  | Log (Total abundance) | 1.71 ± 0.38 | 4.45 | **<0.001** |
| **2.B (NB)** | Intercept | -4.42 ± 0.98 | -4.52 | **<0.001** |
|  | Richness | 0.16 ± 0.04 | 4.05 | **<0.001** |
| **2.C (P)** | Intercept | -11.32 ± 2.25 | -5.04 | **<0.001** |
|  | Log (richness weighted) | 1.36 ± 0.28 | 4.83 | **<0.001** |

**Appendix 9**

Poisson or Negative binomial generalised linear mixed effects models, with a log link, of (1) Skylark, (2) Corn Bunting and (3) Linnet abundance on the Scottish Farmer Cluster versus (A) total specialist bird abundance, (B) specialist bird species richness and (C) specialist bird abundance weighted by specialist bird species richness (richness weighted). Significant values are in bold. The family used in models is indicated by P (Poisson) orNB (Negative binomial).

|  | **Fixed effects** | **Estimate ± SE** | **Z-value** | **P** |
| --- | --- | --- | --- | --- |
| **1.A Skylark (NB)** | Intercept | 2.45 ± 0.53 | 4.59 | **<0.001** |
|  | Log (Specialists) | 0.03 ± 0.09 | 0.29 | 0.77 |
| **1.B (NB)** | Intercept | 2.33 ± 0.58 | 4.02 | **<0.001** |
|  | Specialist richness | 0.04 ± 0.07 | 0.56 | 0.58 |
| **1.C (NB)** | Intercept | 1.37 ± 0.99 | 1.39 | 0.17 |
|  | Log (richness weighted) | 0.21 ± 0.17 | 1.26 | 0.21 |
| **2.A Corn Bunting (P)** | Intercept | -3.32 ± 1.29 | -2.57 | **<0.05** |
|  | Log (Specialists) | 0.84 ± 0.31 | 2.73 | **<0.01** |
| **2.B (P)** | Intercept | -2.10 ± 1.90 | -1.11 | 0.27 |
|  | Specialist richness | 0.27 ± 0.27 | 1.00 | 0.32 |
| **2.C** | Intercept | -7.17 ± 2.88 | -2.49 | **<0.05** |
|  | Log (richness weighted) | 1.22 ± 0.47 | 2.60 | **<0.01** |
| **3.A Linnet (P)** | Intercept | -1.62 ± 0.58 | -2.80 | **<0.01** |
|  | Log (Specialists) | 0.92 ± 0.14 | 6.46 | **<0.001** |
|  | Round (2) | 0.30 ± 0.11 | 2.65 | **<0.01** |
| **3.B (P)** | Intercept | -2.46 ± 1.28 | -1.92 | 0.06 |
|  | Specialist richness | 0.66 ± 0.20 | 3.36 | **<0.001** |
| **3.C (P)** | Intercept | -4.17 ± 1.22 | -3.42 | **<0.001** |
|  | Log (richness weighted) | 1.00 ± 0.20 | 5.01 | **<0.001** |

**Appendix 10**

Poisson generalised linear mixed effects models, with a log link, of Goldfinch abundance on the Scottish Farmer Cluster versus (A) total specialist bird abundance, (B) specialist bird species richness, and (C) specialist bird abundance weighted by specialist bird species richness (richness weighted). Significant values are in bold.

|  | **Fixed effects** | **Estimate ± SE** | **Z-value** | **P** |
| --- | --- | --- | --- | --- |
| **1.A Goldfinch** | Intercept | -3.70 ± 1.68 | -2.20 | **<0.05** |
|  | Log (Specialists) | 0.94 ± 0.34 | 2.76 | **<0.01** |
|  | Round (2) | 0.78 ± 0.42 | 1.86 | 0.06 |
|  | Year (2023) | -0.24 ± 0.46 | -0.52 | 0.60 |
| **1.B** | Intercept | -1.24 ± 0.92 | -1.34 | 0.18 |
|  | Specialist richness | 0.10 ± 0.07 | 2.56 | **<0.05** |
|  | Round (2) | 0.73 ± 0.43 | 1.68 | 0.09 |
| **1.C** | Intercept | -4.74 ± 1.93 | -2.46 | **<0.05** |
|  | Log (richness weighted) | 0.72 ± 0.24 | 2.97 | **<0.01** |
|  | Round (2) | 0.76 ± 0.42 | 1.80 | 0.07 |
|  | Year (2023) | -0.25 ± 0.46 | -0.55 | 0.58 |
